# Feasibility, Added Value, and Radiation Dose of Combined Coronary CT Angiography and Stress Dynamic CT Myocardial Perfusion Imaging in Moderate Coronary Artery Disease: A Real-World Study

**DOI:** 10.3390/jcdd12070241

**Published:** 2025-06-24

**Authors:** Marco Fogante, Enrico Paolini, Fatjon Cela, Paolo Esposto Pirani, Liliana Balardi, Gian Piero Perna, Nicolò Schicchi

**Affiliations:** 1 Maternal-Child, Senological, Cardiological Radiology and Outpatient Ultrasound, Department of Radiological Sciences, University Hospital of Marche, 60126 Ancona, Italy; fatjon.cela@ospedaliriuniti.marche.it (F.C.); paolo.espostopirani@ospedaliriuniti.marche.it (P.E.P.); nicolo.schicchi@ospedaliriuniti.marche.it (N.S.); 2 Cardiology and Intensive Cardiac Care Unit, Cardiovascular Department, University Hospital of Marche, 60126 Ancona, Italy; enrico.paolini@ospedaliriuniti.marche.it (E.P.); gianpiero.perna@ospedaliriuniti.marche.it (G.P.P.); 3 Health Professions Area, Diagnostic Technical Area, University Hospital of Marche, 60126 Ancona, Italy; liliana.balardi@ospedaliriuniti.marche.it; 4 Cardiovascular Radiological Diagnostics, Department of Radiological Sciences, University Hospital of Marche, 60126 Ancona, Italy

**Keywords:** coronary artery disease, coronary stenosis, myocardial perfusion, dynamic CT imaging, CT myocardial perfusion, myocardial blood flow, myocardial blood volume, radiation dose

## Abstract

Objective: We aimed to evaluate the feasibility, added value, and radiation dose of coronary computed tomography angiography (CCTA) and stress dynamic CT myocardial perfusion imaging (MPI) in patients with coronary artery disease (CAD) in a real-world setting. Materials and Methods: This retrospective study included 65 patients (mean age: 51.2 ± 11.5 years; 21 female) with moderate CAD, selected from the Radiological Database of our hospital between May 2022 and December 2024. All patients underwent CCTA and stress dynamic CT-MPI using a third-generation dual-source CT scanner. The shuttle-mode acquisition technique was used for CT-MPI with 60 mL of contrast (iopamidol, 370 mg iodine/mL) administered at a flow rate of 6 mL/s. The mean myocardial blood flow (MBF) and other quantitative parameters were measured for both CAD and reference segments (RSs). A 17-segment-based analysis was employed (excluding the apex). The MBF ratio, defined as the mean MBF value of CAD segments divided by that of RS, was used with a cut-off value of 0.85 to distinguish hypoperfused from non-hypoperfused segments within CAD territories. Non-parametric statistical tests were applied. Results: A total of 1040 segments were evaluated. In 62 segments, the mean MBF of CAD territories was found to have decreased. The mean MBF and myocardial blood volume (MBV) in hypoperfused CAD segments were 65.1 ± 19.8 mL/100 mL/min and 14.5 ± 2.7 mL/100 mL, respectively, both significantly lower compared to non-hypoperfused CAD segments and RSs (*p* < 0.001). The mean effective dose of the protocol was 6.3 ± 1.4 mSv, corresponding to an estimated individual lifetime cancer risk of approximately 0.06% per test, based on BEIR VII Phase 2 modeling. This risk is cumulative, with repeat testing over a 10-year period potentially increasing lifetime cancer risk in proportion to total radiation exposure. The mean total examination time was 26 ± 4 min. Conclusion: The combined CCTA and dynamic CT-MPI protocol is feasible in real-world clinical practice and offers a comprehensive morphological and functional assessment of moderate CAD, with a manageable radiation dose and examination time.

## 1. Introduction

Coronary artery disease (CAD) remains the leading cause of death among adults worldwide [1].

Coronary computed tomography angiography (CCTA) plays a well-recognized diagnostic and prognostic role in patients with a low-to-intermediate risk of CAD [2,3]. This is due to its high sensitivity and negative predictive value in ruling out CAD [4], earning it a Class I designation in the level of evidence according to current European and American guidelines [5,6].

Despite its strengths, identifying moderate CAD using CCTA often poses a challenge for appropriate patient management. Additional tests, such as stress–rest single-photon emission computed tomography (SPECT), are often required to evaluate the functional significance of moderate CAD in myocardial perfusion and to determine the need for invasive coronary angiography [7]. However, SPECT has long acquisition times, poor spatial resolution, and exposes patients to significant radiation doses due to the tracers used [8].

To address these issues, a “one-stop-shop” imaging modality that overcomes these limitations is needed in clinical settings. The recent introduction of dynamic CT myocardial perfusion imaging (MPI) may improve the identification of moderate CAD requiring an invasive treatment approach [9,10]. This technique analyzes pixel attenuation values in the myocardium over time to generate time–activity curves, which are then used to derive perfusion parameters such as peak enhancement, time to peak (TTP), myocardial blood flow (MBF), and myocardial blood volume (MBV) [11].

Dynamic CT-MPI is a valid alternative to SPECT for assessing the functional significance of moderate CAD, offering comparable diagnostic and prognostic value while also improving patient satisfaction [12,13]. Additionally, several studies have demonstrated that its diagnostic accuracy is comparable to other non-invasive gold-standard imaging modalities, such as stress magnetic resonance imaging and positron emission tomography perfusion [14,15]. Agata et al. [16] found a strong correlation between CT-MPI and fractional flow reserve, concluding that CT-MPI reduces the number of unnecessary catheterizations. A recent study by Nous et al. [17] further supports the use of dynamic CT-MPI in clinical practice alongside CCTA.

However, the primary limitation in implementing a protocol combining CCTA and CT-MPI lies in its feasibility in real-world settings, particularly in terms of radiation dose and total examination time.

Therefore, the aim of our study is to evaluate the feasibility, added value, and radiation dose of a protocol combining CCTA and stress dynamic CT-MPI in patients with suspected CAD in a real-life setting.

## 2. Materials and Methods

### 2.1. Study Population

In this retrospective study, patients who underwent CCTA and stress dynamic CT-MPI were selected from our hospital database between May 2022 and December 2024. Inclusion criteria were the presence of moderate CAD, defined as 50–69% stenosis, involving one to three coronary vessels, and the availability of stress SPECT myocardial perfusion imaging performed within three months of the CT examination. The rationale for this dual-imaging approach was to allow internal validation and comparison.

To maintain a homogeneous study population with native coronary artery anatomy, we excluded patients with a history of myocardial infarction, coronary stent implantation, or coronary artery bypass graft surgery.

All patients provided written informed consent, and the study protocol was approved by the Institutional Review Board (ID: 3560—approval date: 20 February 2025).

**Figure 1:**
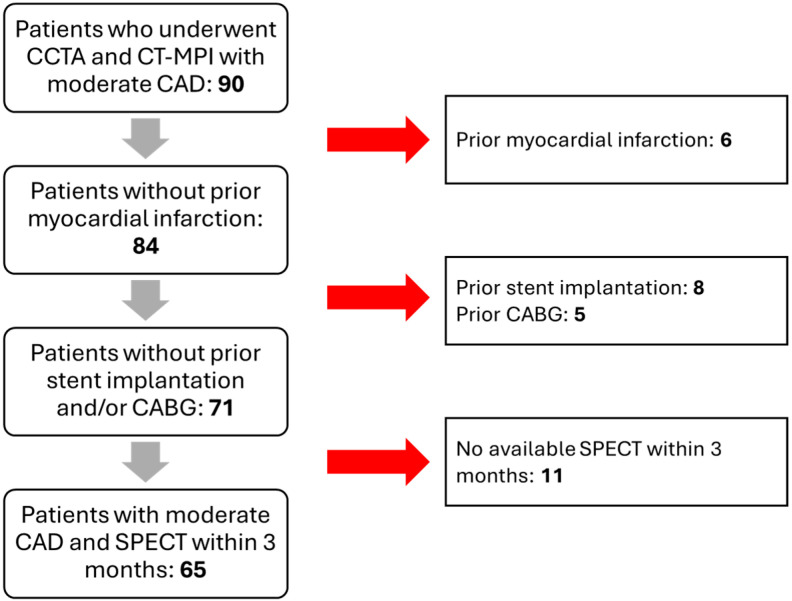
Flow chart of the recruitment process. Abbreviations—CCTA: coronary computed tomography angiography; MPI: myocardial perfusion imaging; CAD: coronary artery disease; CABG: coronary artery bypass graft; SPECT: single-photon emission CT.

The initial study population consisted of 95 patients. However, 6 patients were excluded due to a previous history of myocardial infarction, 8 patients due to prior coronary stent implantation, 5 patients due to previous coronary artery bypass graft surgery, and 11 patients due to the absence of a stress SPECT myocardial perfusion imaging within three months of the CT examination. Therefore, 65 patients were ultimately included in the study (Figure 1).

For each patient, anthropometric features, cardiovascular risk factors, symptoms, heart rate before and after pharmacological stress CT-MPI, radiation dose (expressed as effective dose), and total examination time were collected.

### 2.2. Scan Protocol

CT examinations were performed using a 384-slice (2 × 192) third-generation dual-source CT scanner (SOMATOM Force, Siemens, Munich, Germany). All patients were instructed to avoid caffeine consumption for 24 h prior to the exam. One 20-gauge cannula was inserted into the superficial vein of the right antecubital fossae and connected to a two-way injector.

All patients were examined using the same standardized protocol. First, a non-contrast-enhanced, a prospectively electrocardiogram (ECG)-triggered scan was acquired to determine the scan length for dynamic CT-MPI. Next, CCTA was performed using a prospectively ECG-triggered spiral ultra-high pitch scan for patients with a heart rate ≤65 bpm or a sequential scan for those with a heart rate >65 bpm. A total of 40 mL of iopamidol (370 mg I/mL) was administered at a flow rate of 5.5 mL/s. Finally, stress dynamic CT-MPI was performed.

Regadenoson was administered as a 5 mL bolus over 10 s, followed by a 10 mL saline bolus over 10 s. A 60 mL bolus of contrast medium was then injected into the antecubital vein at a rate of 6 mL/s, followed by a 40 mL saline flush. The dynamic CT-MPI acquisition, using the “shuttle technique,” was initiated 1 min after regadenoson administration, simultaneously with contrast medium infusion.

Cardiac rhythm was continuously monitored, and blood pressure was measured before and after CT-MPI examination. Depending on the heart rate, 10–15 scan phases were acquired over approximately 30 s. All CT scans were performed with automatic tube voltage modulation (CARE kV, Siemens), dose modulation (CAREDose4D, Siemens), and an iterative reconstruction algorithm (Advanced Modeled Iterative Reconstruction, strength 4, Siemens). Table 1 summarizes the scan protocol and parameters.

### 2.3. CCTA Imaging Analysis

The post-processing analysis was performed using dedicated computer-aided evaluation software (syngo.via, version VB10A; Siemens). To evaluate CAD, the clinical application “CT Cardiac” was used. For better delineation of the vessel wall and better evaluation of CAD, two sets of axial images were reconstructed using a third-generation iterative reconstruction technique. This involved a smooth kernel (Bv40, strength 3) and a sharp kernel (Bv44, strength 4).

The coronary artery calcium (CAC) score, presence and severity of coronary stenosis, and plaque characteristics were assessed independently by two radiologists, each with over 10 years of experience in coronary CT angiography. Both radiologists were blinded to each other’s findings during the evaluation. In the event of any discordance, the final decision was made through consensus between the two radiologists.

Coronary arteries were segmented according to the guidelines of the American Heart Association [18]. The CAC score was calculated from the pre-contrast acquisition. Moderate CAD was defined as a luminal diameter stenosis ranging from 50% to 69%, based on quantitative assessment on CCTA. CCTA findings were interpreted according to the Coronary Artery Disease-Reporting And Data System (CAD-RADS) 2.0. Each coronary lesion was graded based on the degree of stenosis and additional modifiers as outlined in the CAD-RADS 2.0 guidelines.

### 2.4. Dynamic CT-MPI Analysis

CT-MPI evaluation involved processing and analyzing images using the dedicated clinical application Myocardial Perfusion Analysis (syngo.via, version VB10A; Siemens). MBF, MBV, and TTP parameters were quantified using a 17-segment model (excluding the apex), with regions of interest placed in cardiac segments corresponding to ≥0.5 cm^3^ of subendocardial myocardium. Measurements were conducted by cardiovascular radiologists with ≥10 years of experience, who were blinded to patients’ medical history and coronary anatomy to assess coronary perfusion defects. Following a previous study [19], the MBF ratio was defined as the mean MBF value of CAD segments divided by the MBF value of all reference segments (RSs). Given the variability in MBF values among patients, an MBF ratio of 0.85 served as the cutoff to distinguish hypoperfused from non-hypoperfused segments. MBF, MBV, and TTP values were quantified in hypoperfused CAD segments, non-hypoperfused CAD segments, and RSs. The two-chamber and four-chamber views of the apical segment were used for quantification. Additionally, the images were processed using a dedicated software application, Cardiac Functional Analysis (syngo.via, version VB10A; Siemens), to automatically segment the left ventricle based on a heart model and generate 17-segment polar maps representing the MBF and MBV distribution.

### 2.5. SPECT Examination

All patients underwent a stress–rest myocardial perfusion imaging protocol using SPECT. Pharmacological stress was induced with adenosine, followed by an intravenous injection of a radiotracer (99mTc-sestamibi or 99mTc-tetrofosmin). Stress images were acquired approximately 30–45 min after tracer administration. Rest imaging was performed on a separate day or after adequate stress recovery, depending on the protocol. Images were reconstructed using standard iterative algorithms, and perfusion defects were visually assessed by two experienced readers blinded to the CT findings.

### 2.6. Statistical Analysis

Statistical analysis was performed by the statistical software MedCalc 14.8.1 (MedCalc Software, Bvba, Oostende, Belgium). Qualitative variables were expressed as number and percentage, while quantitative variables were expressed as mean ± standard deviation.

The sex distribution, symptoms, risk factors, mean age, CAC score, and CAD distribution of the patients were analyzed and calculated. The mean heart rate before and after pharmacological stress, mean radiation dose value, and mean examination time were calculated. The pre-test probability of CAD was evaluated according to the CAC score. The mean MBF_CADsegments_/MBF_RS_, MBV_CADsegments_/MBV_RS_ and TTP_CADsegments_/TTP_RS_ were calculated and compared. The mean MBF, MBV, and TTP value of CAD segments for hypoperfused and non-hypoperfused segments and RSs were calculated and compared.

Mann–Whitney U test was used to compare data for quantitative variables. The chi-square test was used to compare data for qualitative variables. For small sample sizes (e.g., comparisons involving *n* < 5), Fisher’s exact test should be used rather than the chi-square. For multiple pairwise comparisons involving myocardial perfusion parameters (MBF, MBV, and TTP), a Bonferroni correction was applied to adjust for type I error due to multiple testing. A *p*-value < 0.05 was considered to indicate statistically significant differences.

## 3. Results

### 3.1. Study Population

Out of the 65 included patients, 44 (67.7%) were male and 21 (32.3%) were female. The mean age was 51.2 ± 11.5 years. The most common cardiovascular risk factor was hypertension. Other risk factors included hyperlipidemia, smoking, and diabetes. The most common cardiovascular symptom was stable angina, while additional symptoms at clinical presentation included atypical chest pain and ECG abnormalities suggestive of possible ischemia (e.g., including ST-segment depression or elevation ≥1 mm, T-wave inversions, or the onset of new arrhythmias during stress testing).

The mean heart rate before and after pharmacological stress was 66.0 ± 8.5 beats per minute and 86.1 ± 8.0 beats per minute, respectively (*p* < 0.001). The mean radiation dose, expressed as the effective dose (ED), was 6.3 ± 1.4 mSv. The mean examination time was 26 ± 4 min. Detailed demographics are shown in Table 2.

### 3.2. CAC and Pre-Test Probabilty of CAD

The mean CAC score in our study population was 372 ± 98 Agatston units. Based on CAC values, the distribution was as follows: 40% of patients (*n* = 26) had a low CAC score (0–99 Agatston units), 35.4% (*n* = 23) had a moderate score (100–399), and 24.6% (*n* = 16) had a high CAC score (≥400). According to the European Society of Cardiology guidelines, the pre-test probability (PTP) for obstructive CAD was estimated. CAC scoring was subsequently used to refine this risk stratification. Based on this combined approach, 28 patients (43.1%) were classified as having intermediate PTP, while 37 patients (56.9%) fell into the high-PTP category. No patients in the cohort were classified as having low PTP.

### 3.3. CCTA Imaging Analysis and CAD-RADS

In CCTA analysis artifacts were absent from all segments. Among the 65 patients, 64.6% presented with single-vessel disease, 27.7% with two-vessel disease, and 7.7% with three-vessel disease. The left anterior descending artery was the most commonly affected vessel (55.9%), followed by the right coronary artery (25.8%) and the circumflex artery (18.3%).

The plaque analysis in the 65 patients with moderate coronary stenosis revealed several key findings. Based on the degree of stenosis, all patients were categorized as CAD-RADS 3. Additionally, based on modifiers, 33 (50.8%) were found to have mild–moderate plaque burden (CAD-RADS P1-P2), 22 (33.8%) severe (CAD-RAD P3), and 10 (15.4%) extensive (CAD-RADS P4). Moreover, 38.5% of patients were found to have high-risk coronary plaque (HRP).

### 3.4. Dynamic CT-MPI Analysis

In the dynamic CT-MPI analysis, no artifacts were observed in any examination. A total of 1040 myocardial segments were evaluated, with 270 segments (26.0%) assigned to CAD territories based on the presence of moderate CAD, and the remaining 770 segments (74.0%) assigned as RSs.

Based on the MBF_CADsegments_/MBF_RS_, hypoperfused CAD segments were identified. A total of 62 out of 270 CAD segments (22.9%) exhibited myocardial hypoperfusion.

The hypoperfused CAD segments-to-RSs ratio was 0.50 ± 0.08, significantly lower than the ratio observed in non-hypoperfused segments (0.95 ± 0.05, *p* < 0.0001).

The comparison of MBV_CADsegments_/MBV_RS_ between hypoperfused and non-hypoperfused CAD segments revealed statistically significant differences. However, no statistically significant difference was observed in the TTP ratio. These results are summarized in Table 3.

The comparative analysis indicated that mean MBF, MBV, and TTP values in hypoperfused segments were significantly lower than those in non-hypoperfused CAD segments and RSs. Specifically, MBF was 65.1 ± 19.8 mL/100 mL/min in hypoperfused CAD segments, compared to 147.5 ± 28.4 mL/100 mL/min in non-hypoperfused CAD segments and 155.2 ± 24.1 mL/100 mL/min in RS (*p* < 0.0001). Additionally, MBV was 14.5 ± 2.7 mL/100 mL in hypoperfused CAD segments, while it was 21.1 ± 5.9 mL/100 mL in non-hypoperfused CAD segments and 22.0 ± 6.9 mL/100 mL in RS (*p* < 0.0001). A Bonferroni correction for multiple testing was applied across MBF, MBV, and TTP. After correction, all comparisons involving hypoperfused segments remained statistically significant for MBF, MBV, and TTP.

Table 4 summarizes the quantitative parameter comparison between CAD segments and RS.

All patients underwent SPECT within three months of the CT examination. Among the 65 patients, stress dynamic CT-MPI revealed myocardial hypoperfusion in 12 patients (18.5%) despite only moderate coronary stenosis on CCTA. This finding may have influenced clinical decision-making regarding invasive angiography or revascularization.

A strong agreement was observed between stress dynamic CT-MPI and SPECT regarding the identification and localization of hypoperfused myocardial segments. Concordant perfusion defects were observed in 11 of the 12 patients identified as hypoperfused by CT-MPI, with overlapping segmental involvement. In one patient, CT-MPI detected hypoperfusion in additional segments not evident on SPECT. Among the 62 hypoperfused segments identified by CT-MPI, 40.3% (*n* = 25) were located in the septal walls and 27.4% (*n* = 17) in the anterior walls. On vessel-based analysis, perfusion defects were present in four patients with single-vessel disease, six patients with two-vessel disease, and two patients with three-vessel disease.

**Figure 2:**
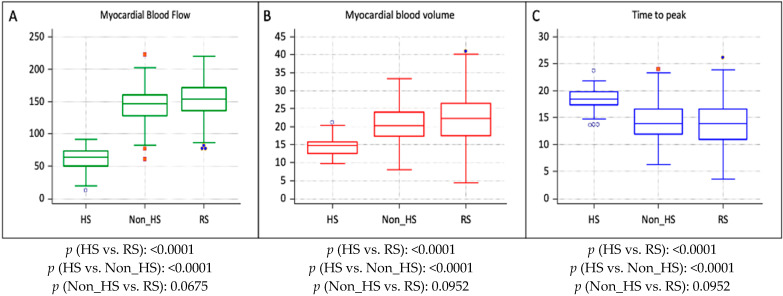
Graphical parameter’s comparison between CAD segments and reference segments. Graphical representation of myocardial blood flow (**A**), myocardial blood volume (**B**), and peak time (**C**) in hypoperfused CAD segment (HS), non-hypoperfused segment (Non_HS), and reference segment (RS).

Figure 2 graphically represents MBF, MBV, and TTP in hypoperfused CAD segments, non-hypoperfused CAD segments, and RSs.

**Figure 3:**
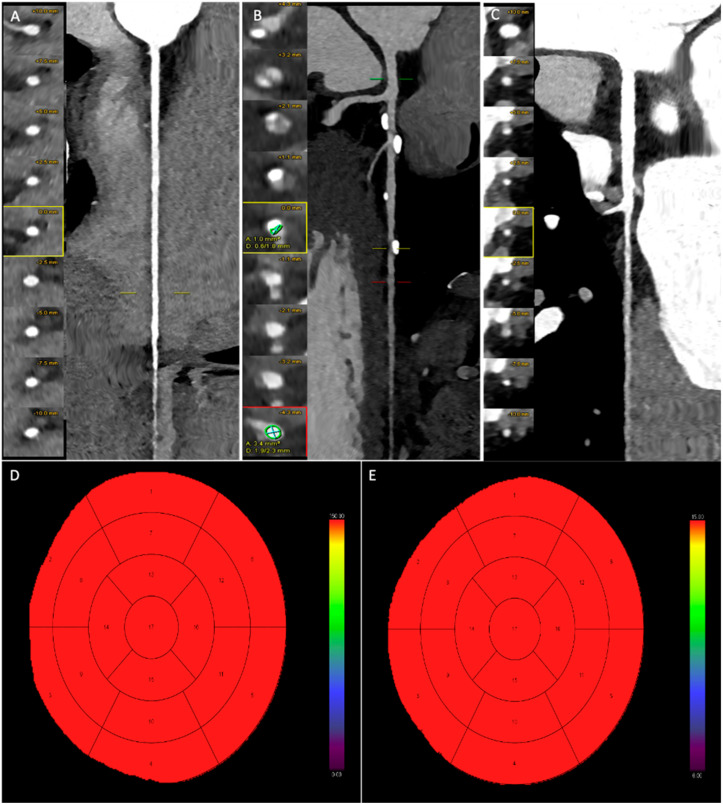
Male patient, 46 years old, with atypical chest pain and ECG abnormalities. Coronary CT angiography examination shows a regularly perfused right coronary artery without significant stenosis (**A**). The left anterior descending artery exhibits multiple mild stenoses (below 40%) at the mid-proximal segment, while a single moderate stenosis (50–69%) is present at the distal segment (**B**). The circumflex artery is regularly perfused without significant stenosis (**C**). (**D**,**E**) Myocardial blood flow (MBF) and myocardial blood volume (MBV) after dynamic stress CT examination, without any reductions in blood flow and volume.

**Figure 4:**
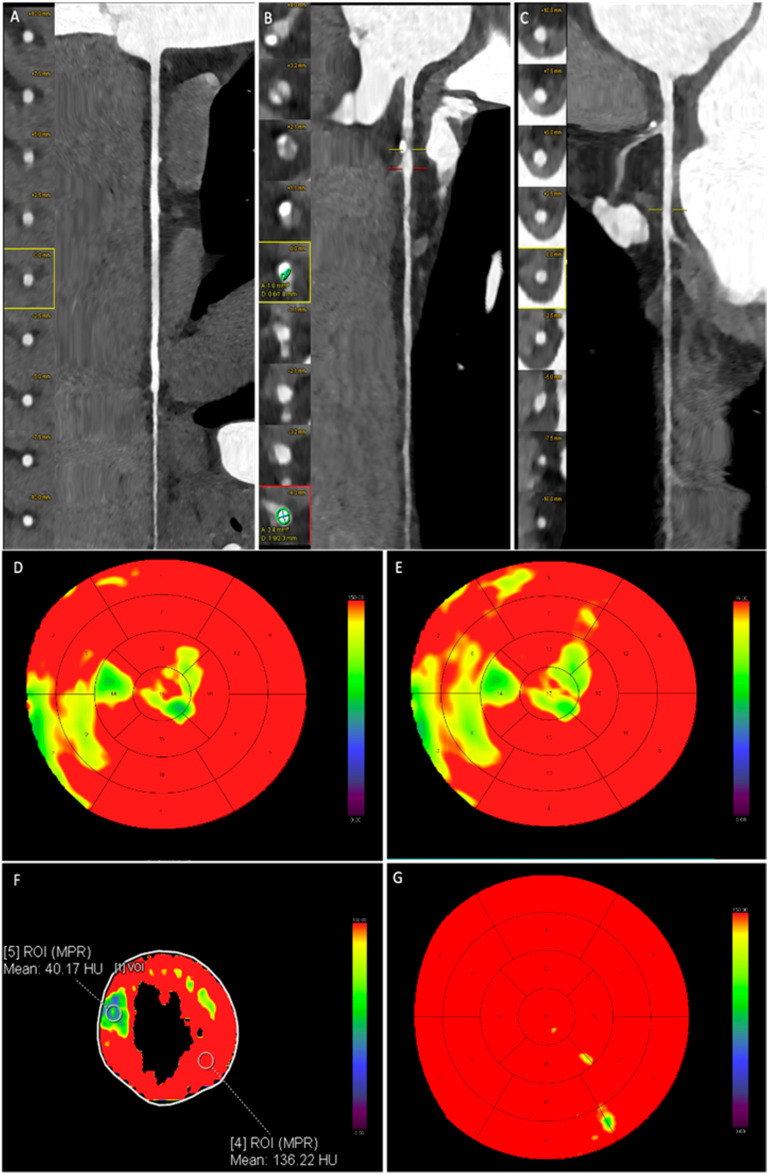
Male patient, 61 years old, with atypical chest pain. Coronary CT angiography reveals a regularly perfused right coronary artery without significant stenosis (**A**). The left anterior descending artery shows a single moderate stenosis with diameter reduction between 50 and 69%, located on a high-risk plaque at the proximal segment (**B**). The circumflex artery is regularly perfused and free from significant stenosis (**C**). Dynamic stress CT myocardial perfusion imaging highlights reduced myocardial perfusion in some basal, mid, and apical septal segments ((**D**,**E**), yellow-green area). In the subsequent post-processing (**F**), the reduction in blood flow is quantitatively assessed by placing a region of interest (ROI) in hypoperfused segments and compared with reference segments. (**G**) shows the normal myocardial vascularization during the baseline evaluation of CCTA.

Figure 3 and Figure 4 present representative cases from our study. Figure 2 depicts a patient with moderate coronary artery stenosis without evidence of myocardial hypoperfusion, whereas Figure 3 illustrates a patient with moderate stenosis associated with stress-induced myocardial hypoperfusion.

## 4. Discussion

CCTA is an effective method for ruling out coronary stenosis in patients with low-to-intermediate CAD probability [5,6]. Since moderate stenoses (50–69%) do not always cause myocardial ischemia, functional tests like SPECT are recommended to assess the need for invasive coronary angiography [20]. However, SPECT has limitations, including long acquisition times, high costs, and significant radiation exposure, and dynamic CT-MPI offers comparable diagnostic accuracy to SPECT and is preferred by patients [12,13,21]. Some studies recommend combining dynamic CT-MPI with CCTA to improve specificity in cases of moderate stenosis [22,23]. Despite its diagnostic potential, the main challenges are radiation dose and examination time. This study aims to evaluate the feasibility, additional value, and radiation dose of this protocol in a real-world clinical setting.

Dynamic CT-MPI enables a precise and quantitative assessment of MBF, MBV and TTP, offering critical insights into both the presence and extent of myocardial ischemia. In our study, significant differences in MBF and MBV were observed between hypoperfused and non-hypoperfused CAD segments, underscoring that moderate coronary stenoses substantially impair myocardial perfusion, not only reducing blood flow but also delaying contrast arrival time. These findings are pivotal because they allow a direct, objective evaluation of the functional impact of coronary lesions, thus informing clinical decision-making regarding the need for revascularization strategies, such as percutaneous coronary intervention or coronary artery bypass grafting [24,25].

Importantly, the integration of stress dynamic CT-MPI into the CCTA protocol provided valuable functional data beyond the purely anatomical assessment of coronary lesions. In our cohort, myocardial hypoperfusion was detected in 16.9% of patients with only moderate coronary stenoses, a finding that could significantly influence patient management by prompting a shift from conservative medical therapy to invasive evaluation and intervention.

Furthermore, the strong agreement observed between stress dynamic CT-MPI and SPECT imaging reinforces the diagnostic reliability of dynamic CT-MPI in detecting functionally significant ischemia. Unlike SPECT, however, CT-MPI offers a higher spatial resolution, shorter acquisition times, and the potential for combined anatomical–functional evaluation within a single session, optimizing workflow and patient comfort.

Our results are consistent with the existing literature. Nous et al. [17] demonstrated the incremental value of dynamic CT-MPI when added to CCTA, improving the identification of hemodynamically significant CAD. Similarly, Nishiyama et al. [9] confirmed that stress CT-MPI significantly enhances diagnostic accuracy compared to CCTA alone, particularly for detecting obstructive CAD that may otherwise be underestimated by anatomical imaging alone.

In our study, we also conducted a detailed assessment of coronary plaque morphology, with particular attention to the identification of “high-risk” plaque features. This analysis underscores the heterogeneous nature of atherosclerotic plaques and their critical role in the pathophysiology and progression of coronary artery stenosis. Recognizing high-risk plaques—characterized by features such as positive remodeling, low-attenuation core, napkin-ring sign, and spotty calcifications—is crucial for more accurate cardiovascular risk stratification [26]. This concept is supported by the reassessment of the PROMISE Trial, which demonstrated that the presence of high-risk plaques on CCTA is independently associated with an increased incidence of major cardiovascular events in patients presenting with typical chest pain [27].

The combined CCTA and stress dynamic CT-MPI protocol demonstrated good applicability in clinical practice, with an acceptable mean radiation dose of 6.3 ± 1.4 mSv. This low exposure is attributable to optimized acquisition strategies, including ultra-high pitch or sequential prospective CCTA protocols [28], a low tube voltage with high tube current settings [29], the use of third-generation dual-source CT with a high temporal resolution and wide coverage [28,30], and a minimized stress CT-MPI acquisition using shuttle mode [31]. This protocol could be particularly useful in addressing diagnostic challenges in younger and athletic populations, where the assessment of coronary stenosis can be more complex and traditional methods may not be sufficient [32]. Compared to previous studies, our protocol achieved a substantially lower dose than that reported by Nishiyama et al. [9] and remained comparable to the range observed with SPECT myocardial perfusion imaging [33]. Conversely, Yang et al. [26] reported a mean estimated radiation dose lower than ours (4.6 mSv) but performed prospectively ECG-triggered high-pitch spiral acquisition for all CCTA exams. Importantly, it allowed comprehensive anatomical and functional coronary assessment in a single session, reducing the need for additional tests [34].

In our study, the overall examination time was 26 ± 4 min. To our knowledge, this is the first study to specifically evaluate the total duration of a combined CCTA and CT-MPI protocol. This information is critical for assessing the feasibility of implementing this approach in clinical practice, particularly given the rising demand for CCTA examinations. Compared to other tests like SPECT, which require longer acquisition times, our protocol could substantially reduce both time and costs, making it a promising alternative, as also suggested by van Assen et al. [35].

The efficiency observed is not only due to the speed of the protocol itself but also to an organized patient management system and, crucially, the involvement of highly trained technical and nursing staff. However, several aspects must be carefully considered when applying this protocol in real-world practice, including staff workload, training requirements, and post-processing times. Regarding staff workload, the procedure should be feasible for existing imaging teams without heavily impacting their current activities. In terms of training, it is essential that the protocol remains accessible to radiologists and technologists with varying levels of experience. Ideally, it should require only minimal additional training, ensuring rapid and seamless integration into routine practice. Post-processing time is another critical factor: if data analysis is too time-consuming, it may undermine the method’s practicality in busy clinical environments. Therefore, streamlined or automated post-processing is highly desirable to enable prompt clinical decision-making.

At our institution, examinations are performed by a team composed of a radiologist specializing in cardiac imaging and a cardiologist who monitors vital parameters and ECG during the procedure. Consequently, close collaboration between radiologists and cardiologists is fundamental for the successful application of this method.

This protocol has potential uses across various clinical settings. For example, it may be particularly valuable in patients with high coronary calcium scores, a known limitation of CCTA alone. Moreover, dynamic CT-MPI was able to detect functionally significant ischemia in patients with CAD-RADS 3, providing incremental diagnostic information beyond anatomical severity alone.

Additionally, some studies have applied dynamic CT-MPI to evaluate stent patency after percutaneous coronary intervention [36], while Schicchi et al. used dynamic perfusion CT to investigate coronary anomalies such as myocardial bridging [8].

In comparison, other modalities such as fractional flow reserve (FFR) CT and dynamic stress cardiac magnetic resonance imaging (CMR) have emerged as powerful non-invasive tools for assessing the hemodynamic significance of coronary stenoses. FFR-CT combines anatomical imaging with computational fluid dynamics, offering high diagnostic accuracy without additional radiation or pharmacological stress. However, its clinical adoption is limited by high costs, limited availability, and dependence on external processing platforms [37]. In contrast, dynamic CT-MPI is performed entirely in-house, offers direct physiological quantification of myocardial blood flow, and is immediately interpretable by the imaging team, making it particularly suited to real-time clinical decision-making in moderate CAD.

Additionally, dynamic stress perfusion CMR represents a robust, radiation-free alternative with excellent spatial and temporal resolution. It has demonstrated high diagnostic accuracy and prognostic value, particularly in intermediate-risk patients. However, its limited availability, longer acquisition times, higher cost, and need for specialized staff and infrastructure restrict its use in many real-world settings [38]. In comparison, CT-MPI offers a more widely accessible and faster solution, especially when anatomical and perfusion data are both required in a single session.

### 4.1. Radiation-Induced Malignancy Risk in Cardiac Imaging

While cardiac imaging techniques such as CCTA and dynamic CT-MPI offer significant diagnostic and prognostic advantages, the radiation burden associated with ionizing modalities warrants careful consideration, particularly in younger or low-risk patients. CCTA alone typically delivers 4–8 mSv, while the addition of stress dynamic CT-MPI increases the dose to approximately 6–10 mSv, consistent with findings in the recent literature. In our study, the mean cumulative dose was 6.3 ± 1.4 mSv, translating per the BEIR VII Phase 2 model to an estimated lifetime attributable cancer risk of 0.06% (1 in 1667) per exam. This risk is cumulative, with repeat testing over a 10-year period potentially increasing lifetime cancer risk in proportion to total radiation exposure. Comparative modalities vary widely: SPECT delivers 9–10 mSv, PET 8–9 mSv, while cardiac MRI and stress echocardiography involve no radiation exposure [39,40,41,42,43].

Despite these differences, in intermediate-risk patients, the value of an integrated anatomic–functional approach can justify the modest radiation burden, particularly when scan protocols are optimized to minimize dose. However, in low-risk or younger patients—especially women—modalities without ionizing radiation may be preferred when clinically appropriate. Ultimately, risk–benefit balance, patient-specific factors, and shared decision-making are key. Radiation risks must not be ignored, but nor should they outweigh the potential diagnostic and therapeutic benefits when the imaging strategy is appropriately selected [44,45,46,47]. Table 5 compares six common cardiac imaging modalities in terms of radiation exposure, cancer risk, and cost.

### 4.2. Limitations to the Study

This study has several limitations. First, it is a retrospective, single-center study; thus, larger multicenter studies are needed to validate these findings. Second, the relatively small sample size may limit the generalizability of the results. As this was designed as a pilot feasibility study, no formal sample size calculation was performed. Third, the absence of consecutive patient enrollment introduces a selection bias, further affecting the generalizability of the results. Fourth, a limitation of this study is the lack of invasive coronary angiography with FFR as a definitive reference standard. However, the study aimed to evaluate real-world feasibility and the diagnostic contribution of CT-MPI in routine practice, where invasive validation is typically reserved for selected cases. Finally, the relatively low prevalence of functionally significant disease in our study population may limit the applicability of the findings. However, this reflects a real-world clinical setting, where many patients referred for CCTA have low-to-intermediate pre-test probability and mainly moderate coronary stenoses. Further larger, multicenter studies involving more diverse populations are necessary to validate our results and confirm their relevance to broader clinical practice.

### 4.3. Clinical Implications

The integration of dynamic CT-MPI with CCTA offers a valuable advancement in the non-invasive assessment of moderate CAD. This protocol combines anatomical and functional evaluation in a single, low-radiation, and rapid imaging session, improving diagnostic accuracy and guiding decisions on invasive procedures or revascularization. By helping avoid unnecessary interventions, it can reduce healthcare costs and is well-suited for high-volume clinical settings. Its ability to assess myocardial perfusion in patients with high coronary calcium scores or post PCI further broadens its clinical utility.

## 5. Conclusions

In conclusion, the combined dynamic CT-MPI and CCTA protocol demonstrates feasibility and provides an enhanced evaluation of moderate CAD in real-world settings, with a manageable radiation dose. This protocol can be successfully integrated into clinical practice. Dynamic CT-MPI plays a crucial role in assessing the hemodynamic impact of coronary stenosis and identifying myocardial ischemia. When paired with CCTA, stress dynamic CT-MPI offers a comprehensive assessment, integrating both anatomical and functional insights. For patients with suspected CAD, this protocol serves as an effective “one-stop-shop,” minimizing the need for additional tests and acting as a gatekeeper for invasive procedures.

## Figures and Tables

**Table 1:** CT scan protocol and parameters.

	First Acquisition CCTA	Second Acquisition Dynamic CTMPI
ECG-protocol	Prospective ECG-triggering set at 35% of the RR interval (HR ≤ 65 bpm) Prospective ECG-gating set from 40% to 60% of the RR interval (HR > 65 bmp)	Prospective shuttle technique acquisition
Scan range	Carina–cardiac apex	Carina–cardiac apex
Pitch	3.2	3.2
Tube voltage	Automatic modulation (CARE kV, Siemens)	70 kV
Tube current	Automatic modulation (CAREDose4D, Siemens)	Automatic modulation (CAREDose4D, Siemens)
Rotation time	0.25	0.25
Temporal resolution	66 ms	66 ms
Collimation	2 × 192 × 0.6 mm	2 × 192 × 0.6 mm
Section thickness/increment	0.6 mm/0.6 mm	3 mm/2 mm
Reconstruction kernel	Body-vascular 40–Body-vascular 44	Body-vascular 40
Iterative reconstruction algorithm	ADMIRE (Siemens)—strength 4	ADMIRE (Siemens)—strength 4

Abbreviations—ECG: electrocardiogram; HR: heart rate; ADMIRE: Advanced Modeled Iterative Reconstruction.

**Table 2:** Sample characteristics.

Sample (*n*)	65
Age (years, mean ± SD)	51.2 ± 11.5
Male (*n*, %)—Female (*n*, %)	44 (67.7)—21 (32.3)
Weight (mean ± SD)	64.8 ± 9.8
Height (mean ± SD)	170.1 ± 13.2
Risk factors	
Hyperlipidemia (*n*, %)	28 (43.1)
Hypertension (*n*, %)	40 (61.5)
Smoking (*n*, %)	19 (29.2)
Diabetes (*n*, %)	16 (24.6)
Clinical presentations	
Stable angina (*n*, %)	38 (58.5)
Atypical chest pain (*n*, %)	24 (36.9)
ECG abnormalities suggestive for possible ischemia (*n*, %)	22 (33.8)
CAC score	
Low (0–99 Agatston units)	26 (40.0%)
Moderate (100–399 Agatston units)	23 (35.4%)
Severe (≥400 Agatston units)	16 (24.6%)
Coronary artery stenosis distribution	
Single-vessel stenosis	42 (64.6%)
Two-vessel stenosis	18 (27.7%)
Three-vessel stenosis	5 (7.7%)
Coronary artery stenosis	
LAD (*n*, %)	52 (55.9%)
CX (*n*, %)	17 (18.3%)
RCA (*n*, %)	24 (25.8%)
CAD-RADS	
Plaque burden (P1, P2, P3, P4)	15 (23.1%), 18 (27.8%), 22 (33.%), 10 (15.4%)
High-risk plaque (*n*, %)	25 (38.5%)

Abbreviations—SD: standard deviation; ECG: electrocardiogram; LAD: left anterior descending artery; CX: circumflex artery; RCA: right coronary artery; CAD-RADS: Coronary Artery Disease-Reporting And Data System; P1: mild; P2: moderate; P3: severe; P4 extensive.

**Table 3:** Ratios of MBF, MBV, and TTP between hypoperfused and non-hypoperfused CAD segments.

Ratios	Hypoperfused CAD Segments (*n* = 62)	Non-Hypoperfused CAD Segments (*n* = 208)	*p*
MBF_CADsegments_/MBF_RS_	0.50 ± 0.08	0.95 ± 0.05	<0.0001
MBV_CADsegments_/MBV_RS_	0.65 ± 0.13	0.93 ± 0.11	<0.0001
TTP_CADsegments_/TTP_RS_	1.10 ± 0.22	1.05 ± 0.11	0.1150

Abbreviations—MBF: myocardial blood flow; MBV: myocardial blood volume; TTP: time to peak; CAD: coronary artery disease.

**Table 4:** Quantitative myocardial perfusion parameters in CAD and reference segments.

Parameters	HS (*n* = 62)	Non_HS (*n* = 208)	RS (*n* = 770)	*p* (HS vs. RS)	*p* (HS vs. Non_HS)	*p* (Non_HS vs. RS)
MBF (mL/100 mL/min)	65.1 ± 19.8	147.5 ± 28.4	155.2 ± 24.1	<0.0001	<0.0001	0.0675
MBV (mL/100 mL)	14.5 ± 2.7	21.1 ± 5.9	22.0 ± 6.9	<0.0001	<0.0001	0.0952
TTP (s)	18.5 ± 3.5	13.7 ± 3.3	14.0 ± 3.6	0.0012	0.0014	0.0870

Abbreviations—RS: reference segments; HS: hypoperfused segments; MBF: myocardial blood flow; MBV: myocardial blood volume; TTP: time to peak; CAD: coronary artery disease; RS: reference segments.

**Table 5:** Radiation exposure, cancer risk, and cost of cardiac imaging modalities.

Modality	Effective Dose (mSv)	Chest X-Rays Equivalent	Cancer Risk Estimate (BEIR VII Phase 2 Model: 0.01%/mSv)	Cumulative Risk Over 10 Years	Estimated Cost (USD)
Cardiac CT	4–8	200–400	0.04–0.08% (1 in 2500–1250)	~0.1% (2 studies)	$400–$500
Cardiac CT + CT Perfusion	6–10	300–500	0.06–0.1% (1 in 1667–1000)	~0.2% (2 scans)	$700–$800
SPECT (Sestamibi)	9–10	450–500	0.09–0.1% (1 in 1111–1000)	~0.2% (2 scans)	$1000–$1500
PET	8–9	400–450	0.08–0.09% (1 in 2500–1111)	~0.1% (2 scans)	$2000–$3000
Cardiac MRI	0	0	0%	0%	$700–$900
Stress Echo (TTE)	0	0	0%	0%	$300–$600

Abbreviations—CT: computed tomography; SPECT: single-photon emission computed tomography; PET: Positron Emission Tomography; MRI: magnetic resonance imaging; TTE: transthoracic echocardiography.

## Data Availability

Data are contained within the article.
